# Revealing the Intricate Effect of Collaboration on Innovation

**DOI:** 10.1371/journal.pone.0121973

**Published:** 2015-03-23

**Authors:** Hiroyasu Inoue, Yang-Yu Liu

**Affiliations:** 1 Department of Business Administration, Osaka Sangyo University, Daito, Osaka, Japan; 2 Channing Division of Network Medicine, Brigham and Women’s Hospital and Harvard Medical School, Boston, MA, United States; CNRS UMR7622 & University Paris 6 Pierre-et-Marie-Curie, FRANCE

## Abstract

We studied the Japan and U.S. patent records of several decades to demonstrate the effect of collaboration on innovation. We found that statistically inventor teams slightly outperform solo inventors while company teams perform equally well as solo companies. By tracking the performance record of individual teams, we found that inventor teams’ performance generally degrades with more repeat collaborations. Though company teams’ performance displays strongly bursty behavior, long-term collaboration does not significantly help innovation. To systematically study the effect of repeat collaboration, we defined the repeat collaboration number of a team as the average number of collaborations over all the teammate pairs. We found that mild repeat collaboration improves the performance of Japanese inventor teams and U.S. company teams. Yet, excessive repeat collaboration does not significantly help innovation at both the inventor and company levels in both countries. To control for unobserved heterogeneity, we performed a detailed regression analysis and the results were consistent with our simple observations. The presented results revealed the intricate effect of collaboration on innovation, which may also be observed in other creative projects.

## Introduction

Collaboration is key to innovation. Indeed, collaboration increases the chances of combinations among ideas, which may result in an innovative and gifted product [1]. For example, an inventor might combine his or her half idea with another inventor’s half idea to realize a whole innovative one. Moreover, collaboration can speed up the delivery of innovations [2], which may involve the parallel validation of initial conceptions and the series implementation of final ideas. Since speed is the last great competitive advantage to innovations, the speed-up gained through collaboration could be a crucial determinant in creative enterprises. While collaboration has been considered as a central theme to innovation, how the effect of collaboration on innovation changes with time has not been quantitatively studied in a systematic fashion.

Previous studies found that repeat collaborations usually underperform in creative projects, e.g., scientific research [3, 4], consulting practice [5], and entertainment performances [3, 6, 7, 8]. Those interesting results were explained by the suppression of “creative abrasion” (a sequence of processes constituted by idea generation, disclosure/advocacy, and convergence), which is key to creative project performance [9]. Despite those intriguing results on the negative relationship between repeat collaboration and team performance, the effect of repeat collaboration on innovation has not been fully understood.

Here we studied the Japan and U.S. patent records of several decades [10, 11, 12] to demonstrate the effect of collaboration on innovation. Patent records are valuable for this research. First of all, the purpose of patents is to facilitate and encourage disclosure of innovations into the public domain for the common good. A typical patent application must meet the relevant patentability requirements such as novelty and non-obviousness. Hence, patent records are directly related to the occurrence of innovations over time [13, 14, 15]. We can track and analyze the innovation activity over long periods of time by mining patent records, in line with the current quantitative trend of computational social science [16]. Comparing with patent records, team performance in scientific research, consulting practice, and entertainment industries, cannot always be directly related to innovation. For example, scientific findings, especially from fundamental sciences, do not always lead to more effective products or technologies that are readily available to markets and society. Second, there are two levels of collaboration in patent records. A patent application can be filed by multiple inventors and/or multiple companies. Though different companies could have different climates and unique tacit knowledge [17], to speed up innovations companies capitalize on other companies’ knowledge more and more [18, 19, 20]. Commensurate with this trend, the number of joint patents applied for by multiple companies keeps increasing those days [21]. Since innovations can be driven by the collaborations of inventors and/or companies, it would be very interesting to study the effect of collaboration on innovation at both the inventor and company levels.

## Collaboration Networks

A patent can be requested by filing an application. The applicants of a patent are inventors or companies. In this work, we analyzed the Japan and U.S. patent records (accessible from http://www.iip.or.jp/e/ and http://www.nber.org/, respectively), which cover different years and different number of inventors and companies.

**Fig 1:**
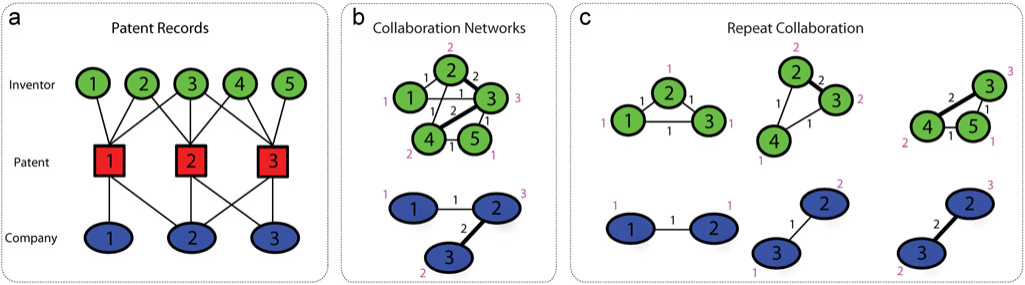
Patent records and the associated collaboration networks. (a) Patent records contain collaborations at both the inventor and company levels. (b) By drawing a undirected link between nodes *i* and *j* if they filed a patent application at least once, we can construct the collaboration networks of inventors (or companies). The total times of collaborations between nodes *i* and *j* over the whole patent record is defined to be the weight of the link (*i*, *j*) (shown in black). The total number of collaborators of nodes *i* over the whole patent record is defined to be the weight of the node *i* (shown in pink). (c) The inventors (or companies) listed in each patent record forms a clique. For each patent, we calculated its repeat collaboration number (*R*_l_) of its inventors (or companies) by averaging the accumulated number of collaborations among all the inventor (or company) pairs in the team (shown in black). The productivity of node *i* in a patent is defined to be the accumulated number of patents that node *i* has contributed. We calculated the team productivity (*R*_n_) by averaging the productivity of all its nodes (shown in pink).

We first studied the structure of the underlying collaboration networks to check the similarity and/or difference of the two patent records. We constructed a collaboration network of inventors (or companies) by drawing a link between two nodes *i* and *j* if they collaborated at least once, i.e., they filed at least one patent application together (see Fig. 1), where nodes are inventors (or companies) and links represent the collaborations between inventors (or companies) [22]. The total number of collaborators of node *i* is called its degree, denoted as *k*_*i*_. The total times of collaborations between nodes *i* and *j* is defined to be the weight of the link (*i*, *j*), denoted as *w*_l_(*i*, *j*). The total number of patents that node *i* has contributed is defined to be its weight, denoted as *w*_n_(*i*).

Table 1 shows the basic information of the Japan and U.S. patent records and the constructed collaboration networks. We found that at the inventor level both Japan and U.S. collaboration networks show very high clustering coefficient *C* and high assortative degree correlations *r*. High *C* indicates that inventors tend to cluster together, i.e., two collaborators of an inventor also tend to be collaborators of each other. High *r* means that hub inventors (with high degree *k*) tend to collaborate with other hub inventors. At the company level, however, both Japan and U.S. collaboration networks display very low clustering coefficient (*C* ≈ 0) and slightly disassortative degree correlations (*r* ⪅ 0), which are qualitatively different from the inventor collaboration networks.

**Table 1:** Patent records and collaboration networks used in this paper. The collaboration networks at the inventor and company levels were constructed from the Japan and U.S. patent records of several decades, with number of patents denoted by *N*_P_. For each collaboration network we show the number of nodes (*N*), edges (*L*), mean degree (⟨*k*⟩ = 2*L*/*N*), relative size of the largest connected component (*s*_lc_ = *N*_lc_/*N*, where *N*_lc_ is the number of nodes in the largest connected component), fraction of isolated nodes (*n*_0_), clustering coefficient (*C*) and degree correlation (*r*). In graph theory, the connected components of a graph *G* are the set of largest subgraphs of *G* that are each connected (i.e., any two vertices or nodes in a connected component are connected to each other by paths), which can be easily computed using either breadth-first search or depth-first search. The largest connected component (often referred to as the giant component) is the connected component of the largest size (number of nodes). The clustering coefficient *C* of a graph is a measure of the degree to which the nodes in the graph tend to cluster together (i.e., form triangles) [23]. *C* can be calculated as (3 × number of triangles)/(number of connected triples). The degree correlation *r* of a network is given by the Pearson correlation coefficient of degrees between pairs of linked nodes [24].

	Patent record		Inventor network							Company network						
	Duration	*N*_P_	*N*	*L*	⟨*k*⟩	*s*_lc_	*n*_0_	*C*	*r*	*N*	*L*	⟨*k*⟩	*s*_lc_	*n*_0_	*C*	*r*
Japan	1994–2008	1,967,361	1,806,259	3,458,690	3.830	0.358	0.135	0.438	0.333	72,840	70,702	1.941	0.364	0.542	0.068	-0.056
USA	1963–1999	2,923,922	1,528,610	2,599,540	3.401	0.453	0.232	0.334	0.151	148,220	15,896	0.214	0.049	0.907	0.041	-0.032

Despite the fact that Japan and U.S. collaboration networks cover different years and different number of inventors and companies, we found that their degree distributions *P*(*k*), node weight distributions *P*(*w*_n_), link weight distributions *P*(*w*_l_), and component size distributions *P*(*S*) display qualitatively similar features (see S2 Fig. for details). At the company level, we found the Japan and U.S. collaboration networks display some quantitative differences. For example, they have different fractions of isolated nodes, denoted as *n*_0_, who never collaborate with others. We found that *n*_0_ = 0.542 for Japan and 0.907 for U.S. Their relative sizes of the largest connected component, denoted as *s*_lc_, are also different: *s*_lc_ = 0.364 for Japan and 0.049 for U.S. The large value of *s*_lc_ in the Japan company collaboration network indicates that Japanese companies are highly connected through collaboration. In contrast, the U.S. companies are not well connected in innovative teams. This structure difference is also reflected by their mean degrees (⟨*k*⟩ = 1.941 for Japan and 0.214 for U.S.). The high value of *n*_0_, low values of *s*_lc_ and ⟨*k*⟩ for the U.S. company collaboration network implies that company collaborations in innovations are not very popular in U.S. Note that according to both the U.S. patent laws (35 U.S.C. 262) and Japanese patent law (Article 73), a company cannot sell or license a jointly applied patent without the consent of others. Yet, Japanese companies seems to be more open to collaborate on patents than companies of the U.S.

## Collaboration and Innovation

### Effect of Team Size

**Fig 2:**
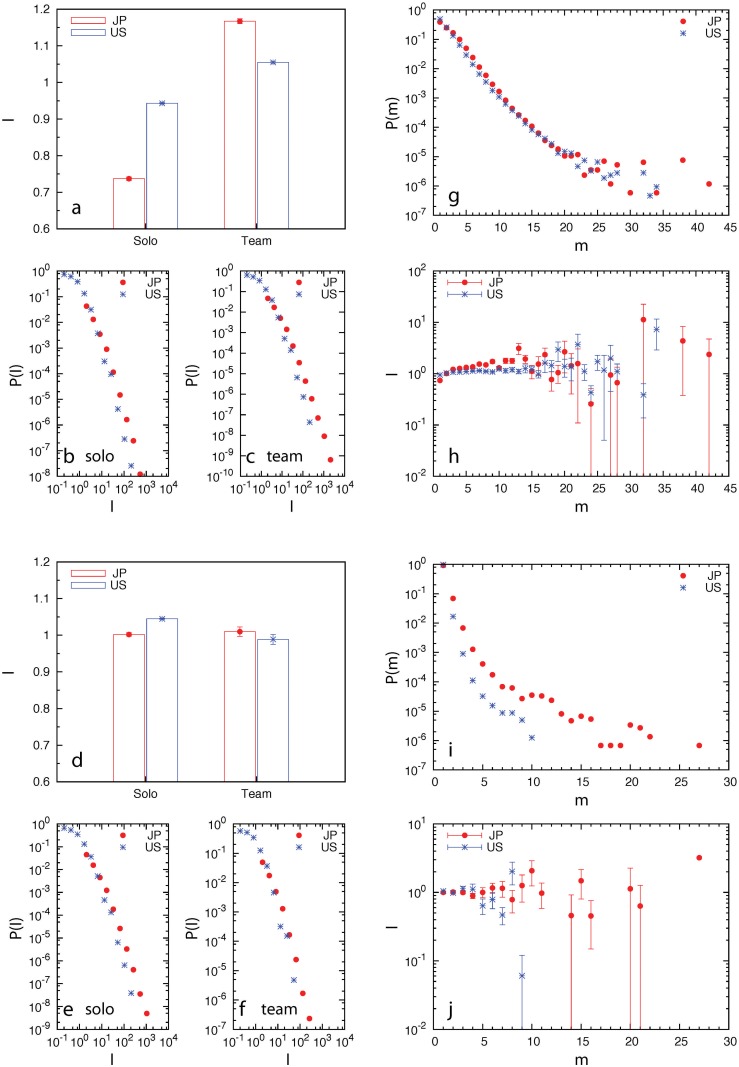
Effect of team size on innovation performance. (a, b, c, g, h) Inventors. (d, e, f, i, j) Companies. (a, d) The average impacts of patents filed by solos and teams. (b, c, e, f) The impact distribution of patents filed by solos (*m* = 1) and teams (*m* ≥ 2). (g, i) The team size distribution. (h, j) The patent impact as a function of team size.

We first illustrated the effect of team size on innovation. Previous studies have shown that inventor teams typically produce more successful patents than solo inventors do [25, 26]. Yet, it is still unknown whether company teams will also outperform solo companies. We denoted the number of inventors or companies listed in a patent record as *m*. An inventor or company team is defined as having more than one listed inventor or company in a patent record (i.e., *m* ≥ 2). To quantify the innovation performance of inventors and companies, we define the impact (*I*) of a patent to be the number of citations of that patent (with self-citations removed) normalized by the average number of citations of patents granted in the same year [27, 11]. We found that on average inventor teams outperform solo inventors (see Fig. 2a), consistent with previous result [25]. However, at the company level, teams do not outperform solos at all (see Fig. 2d). In fact, the average patent impact of the U.S. company teams is even less than that of U.S. solo companies. To further compare the performance of solos and teams, we calculate the impact distributions *P*(*I*) of patents invented by solos and teams, separately (Fig. 2b, c, e, f). We found that *P*(*I*) displays fat-tailed distributions at both the inventor and company levels, consistent with the result of *P*(*I*) calculated for all patent records regardless of whether they were filed by inventors or companies (see S3 Fig.).

To reveal more information about the effect of team size on innovation, we systematically studied the patent impact (*I*) as a function of team size (*m*) (Fig. 2h, j). We found that the team performance, as measured by the impact of their patents, behaves differently at the two different levels as the team size increases. For inventor teams, the patent impact increases slowly as team size *m* increases (up to *m* ≈ 15), especially for the Japanese inventor teams, consistent with the results shown in Fig. 2a. For company teams, however, the patent impact does not increase significantly with increasing *m*, consistent with the results shown in Fig. 2d. We also noticed that for both inventor and company teams their performance displays large fluctuations with large team size *m*, which could be due to the fact that large teams are rather rare in both Japan and U.S. patent records (see Fig. 2g, m).

### Effect of Team Experience

#### Repeat Collaboration

**Fig 3:**
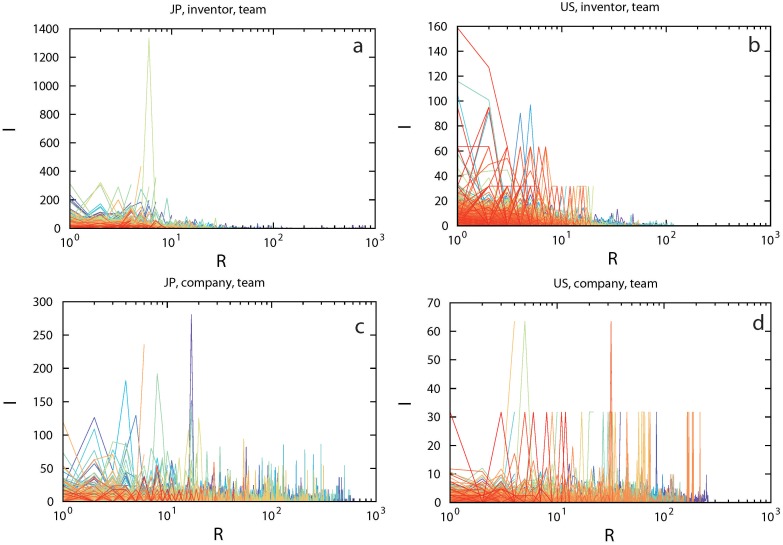
Track records of individual teams with at least three patent records. Different colors represent different individual teams. (a, b) Inventors. (c, d) Companies.

Team experience is an important factor that could potentially affect a team’s innovation performance. To demonstrate the effect of team experience on innovation, one can simply track the performance of each team. For a given team, represented by a set of inventors or companies, we define its *exact repetition number* (*R*) as the accumulated number of patent applications that the whole team has filed together up to the current patent. We then label teams according to their inventor or company set and track each team’s performance by plotting the impact of their patents as a function of *R* (see Fig. 3). We found that extremely successful patents (indicated by their huge impact) are typically among the first 10 patents of most inventor teams. For company teams, their patent records display many impact spikes, indicating that individual company teams occasionally perform extremely well.

In the above analysis, we focused on the repeat collaboration of the whole team rather than its members. Before the whole team work together again, some of its team members may have already collaborated or worked alone on some other patents. To take this into account and systematically study the effect of repeat collaboration on innovation using all the patent records of teams, we denote *R*_*ij*_ of a node pair (*i*, *j*) in a patent record as the accumulated number of repeat collaborations between *i* and *j* up to that patent. We then define the *repeat collaboration number* (*R*_l_) of a team listed in a patent record as the average *R*_*ij*_ of all its teammate pairs. Note that for any given patent, the repeat collaboration number of the inventor team and that of the company team are generally different. For example, in Fig. 1, for patent-2, *R*_l_ = (1+1+2)/3 = 4/3 for the inventor team while *R*_l_ = 1 for the company team.

**Fig 4:**
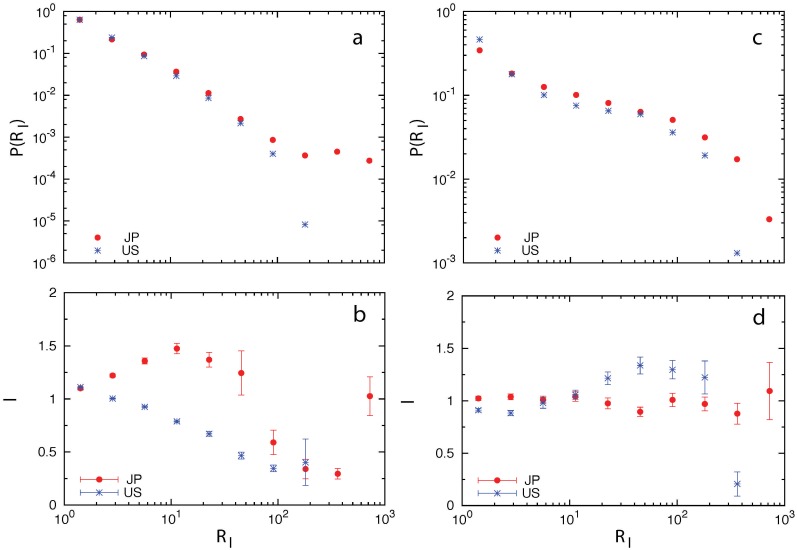
Effect of repeat collaboration on innovation performance. (a, b) Inventors. (c, d) Companies. (a, c) Probability distributions of repeat collaboration number of teams. (b, d) Patent impact as a function of repeat collaboration number.

For each patent in the patent records of teams, we calculated its *R*_l_ and found that *R*_l_ shows a broad distribution for both inventor and company teams (see Fig. 4a, c). We then calculated the average patent impact for teams of similar *R*_l_ grouped in logarithmic bins (see Fig. 4b, d). We found that the effects of repeat collaboration at the inventor and company levels are qualitatively different. At the inventor level, we found Japanese teams and U.S. teams also display quite different behavior. The innovation performance of Japanese inventor teams improves first as *R*_l_ increases, reaches its peak value at *R*_l_ = 10, and then generally degrades for *R*_l_ > 10 (except the abnormal behavior around *R*_l_ ≈ 700, where the patent impacts increases but is still not significantly higher than that of teams with *R*_l_ < 10.) This suggests an ideal timing for Japanese inventors to make new collaborations and hence “rejuvenate” the inventor team. In contrast, the performance of U.S. inventor teams degrades almost monotonically as *R*_l_ increases, implying that repeat collaborations weakens the creativity of U.S. inventor teams. At the company level, Japanese teams show remarkably stable performance for *R*_l_ up to 10^3^. For U.S. company teams, their performance slightly improves as *R*_l_ increases up to 100 and then degrades with increasing *R*_l_.

#### Regression Analysis

Besides the exact repetition number (*R*) and the repeat collaboration number (*R*_l_) of a team, there are numerous variables related to team experience, e.g., *team age* (denoted as *A*, the average of the team members’ “age”, i.e., the duration from its first application year to the current application year), *team productivity* (denoted as *R*_n_, the average number of patents that inventors/companies of a team have already applied), etc (See S1 Table. The table shows variables related to team experience and other variables). To control for unobserved heterogeneity, we performed a detailed regression analysis to investigate the effects of those team experience variables [26].

We introduced a variable for an offset term, i.e., cited_count_average (the average citation of patents applied in the same year as patent *i*) such that the response variable (cited_count, i.e., the number of citations patent *i* obtained) is closely related to the definition of a patent’s impact. We also introduced longevity as a control variable. We have checked correlations of the variables and what regression analyses we can conduct (See S2–S5 Tables). We didn’t use breadth_of_search as a control variable because it strongly correlates with the variable patent_references. All the explanatory variables are the variables closely related to team experience, i.e., inventor_age (or company_age), team_productivity, etc.

We adopted a generalized linear mixed model [28]

$$\begin{array}{c} \lambda_{i}=\exp\left[\beta_{0}+\sum_{k}\beta_{k}x_{ki}+\log\left(\mathrm{cited}\_\mathrm{count}\_\mathrm{average}\_i\right)+r_{i}\right] \end{array}$$ (1)

where *λ*_*i*_ is the response variable, i.e., its the citation count, *β*_0_ is the intercept, *β*_*k*_ is the coefficient for the *k*-th variable, *x*_*ki*_ is the *k*-th variable for patent *i*, *r*_*i*_ denotes random noise following a gamma distribution.

We found that at the inventor level all the control variables have significant coefficients (See S6 and S7 Tables) and the results are consistent with previous work [26]. For example, the control variable team_size is significant and has positive coefficient for both Japanese and U.S. inventors (consistent with the result shown in Fig. 2a). At the company level, some of the control variables did not have significant coefficients in some data sets (See S8 and S9 Tables). For examples, the control variable team_size of Japanese companies is not significant (consistent with the result shown in Fig. 2d); the control variable, longevity of Japanese companies is not significant either.

One important perspective of the regression analyses is to compare the accountability of those explanatory variables. To do that, we checked the relative goodness of fit of seven statistical models including different sets of variables:

(1) base model, which contains the following control variables: {team_size, unassigned, university, claims, patent_reference, and longevity}.
(2) base model + team_age (inventor_age or company_age).
(3) base model + team_patents (*R*).
(4) base model + team_experience_diversity.
(5) base model + team_network_size.
(6) base model + team_productivity (*R*_n_).
(7) base model + repeat_collaboration_number (*R*_l_).

For each statistical model, we calculated its Akaike information criterion (AIC) value. A model with lower AIC value means that it can fit data better. We exploited the difference of AIC in models to compare the team experience variables. We found that the performance of the statistical models with different explanatory variables varies a lot in the sense that their AIC values heavily depend on the dataset. See bottoms of S6–S9 Tables. Since each dataset has different number of samples, AIC values are largely different between datasets. Yet, we found the models (2) and (7) display relatively stable performance in the sense that these two models always have lower AIC values than the base model. This indicates that team_age (*A*) and repeat_collaboration_number (*R*_l_) are better than other explanatory variables in explaining the data sets.

Moreover, coefficients of *A* and *R*_l_ are negative for all data sets, except that the coefficient of *R*_l_ for U.S. company data set is slightly positive. Note that the small positive coefficient of *R*_l_ for U.S. company data set is consistent with the result shown in Fig. 4d, where we observed that U.S. company teams’ performance slightly improves as *R*_l_ increases up to 100 and then degrades as *R*_l_ increases further.

We have tested the control variables and one of the explanatory variables. It is also possible to compare these variables simultaneously by using automatic variable selection algorithms, e.g., the least absolute shrinkage and selection operator (LASSO) method [29]. The results of LASSO were shown in S10 Table. We acquired the order of variables according to their importance. The best models have minimum mean-squared errors. We found the followings. (1) There were no deciding explanatory variables because the orders of them were different in comparing the inventor (or company) datasets. (2) *A* and *R*_l_ had relatively higher priorities than other explanatory variables. (3) Japanese inventor and company datasets did not converge. However, U.S. inventor and company datasets did converge, and *A*, *R*_n_, and *R*_l_ were included in the converged models. Based on these findings, we argued that age (*A*) and repeat_collaboration_number (*R*_l_) are certainly related to the impact of patents.

#### Interplay between Team Age and Repeat Collaboration

**Fig 5:**
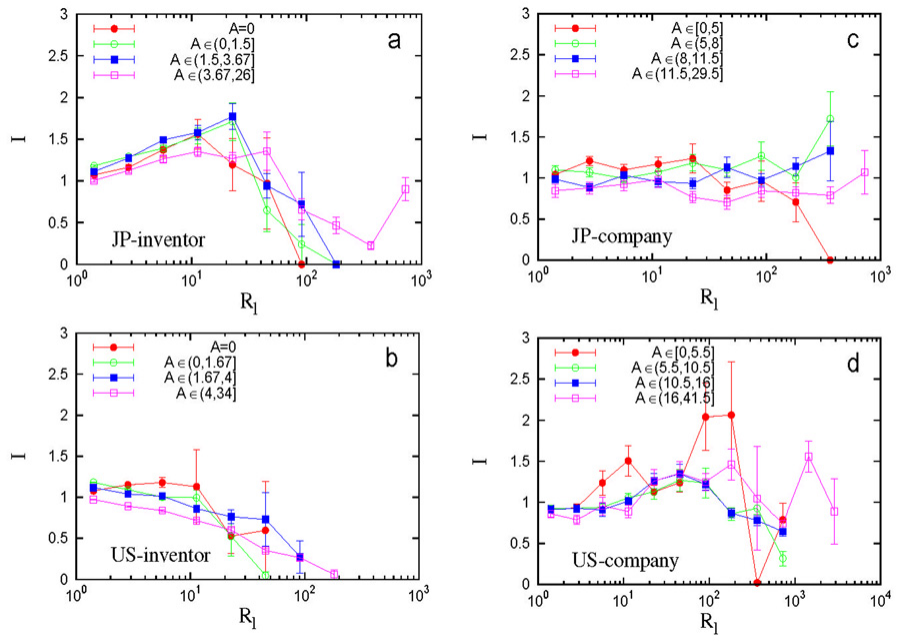
Effect of repeat collaboration on innovation performance of teams with similar team age. (a, b) Inventors. (c, d) Companies. To separate the aging effect of a team from that of repeat collaboration among its teammates, we grouped patents according to the quartiles of their team age (*A*). The *A*-range of each group is shown in the legend. For each *A*-group we further grouped patents according to their repeat collaboration number (*R*_l_) and then calculated the average patent impact for each *R*_l_-subgroup.

The result of regression analysis prompts us to study the interplay between *A* and *R*_l_, i.e., the “aging” of team members and the repeat collaboration among them. Naturally the degradation of team performance with large *R*_l_ could be possibly due to the fact that when teams become older (i.e., *A* is very large) they are less innovative. (Actually, *A* shows similar features to *R*_l_. See S5 Fig. for more information.) To address this issue and further reveal the intricate effect of collaboration on innovation, we grouped patents according to quartiles of their team age and then for each group we calculated the patent impact as a function of *R*_l_ (see Fig. 5). Now within each group the change of innovation performance is mainly due to the repeat collaborations quantified by *R*_l_. At the inventor level, we found that for Japanese inventor teams, regardless of their team age, their performance improves first as *R*_l_ increases and then degrades. U.S. inventor teams’ performance degrades almost monotonically with increasing *R*_l_, regardless of their team age. At the company level, we found that Japanese company teams of different team age show remarkably stable innovation performance as *R*_l_ increases. In contrast, U.S. company teams’ performance displays quite unstable behavior and there is no significant improvement for large *R*_l_, regardless of the team age.

## Discussion

Though for Japanese inventor teams and U.S. company teams a moderate repeat collaboration slightly improves their innovation performance, we did not find strongly positive relationship between innovation and collaborations in the long run. Current results actually suggest that there is a negative relationship between them, especially at the inventor level and for long term collaboration.

At the inventor level, we observed that Japanese inventor teams have a performance peak around repeat collaboration number *R*_l_ ≈ 10, while for U.S. inventor teams their innovation performance drops almost monotonically as *R*_l_ increases. This result raises an interesting question worthy of future pursuit: What causes the drastically different effects of repeat collaboration on the performance of the two countries’ inventor teams? We leave the systematic study of this question as future work. Here we want to point out the different innovation climates in U.S. and Japan, which might help us better understand this question. Typically, U.S. workers are subject to the strong pressure/incentive for the immediate result and light regulations from the labor market [30], implying that taking time for U.S. inventors to deepen their collaborations is not a good strategy. In contrast, the labor regulation of Japan is strict and a group of individuals can create value among them due to cohesive culture [31].

At the company level, we observed that Japanese company teams display remarkably stable innovation performance while U.S. company teams slightly outperform with repeat collaborations up to some point. This might be related to the fact that in U.S. joint patents of companies are still not very common, while in Japan joint patents of companies have been prevailing. A deeper understanding deserves a systematic study in the future. Here we emphasize that the difference of intercompany relationships in the two countries could be useful to explain the observation. In Japan there is a unique company ties called “Keiretsu”, i.e., a set of companies with interlocking business relationships and shareholdings and hence they typically share human assets and information [32]. Since “Keiretsu” significantly eliminates the difference of inter-company and inner-company, there may be no margin to deepen their collaborations on innovation. In contrast, U.S. companies do not have such prior connections and hence they could build deeper collaborations as they have more joint patents. Consequently, the longitudinal relationship of U.S. companies nurture the trust and therefore better performance [33]. Yet, in the long run, overembeddedness limits the diversity of information and hence stifles the creation [34]. This may explain the non-monotonic behavior of the innovation performance of U.S. company teams.

The results presented here provide us a novel perspective about the strategy of improving innovation performance via controlling the repeat collaboration number at inventor or/and company levels. For example, our results suggested that repeat collaborations of Japanese companies have very stable performance while repeat collaborations of Japanese inventors have non-monotonic effect on their performance. Hence, Japanese companies should carefully monitor the repeat collaboration number (*R*_l_) of their inventor teams (rather than that of their company teams) and force the inventors to make new collaborations when *R*_l_ is close to 10. On the other hand, U.S. inventor teams’ performance degrades monotonically with more repeat collaborations while U.S. company teams’ performance have a peak value around *R*_l_ ≈ 100. Hence, U.S. companies should proactively collaborate with other companies but change practitioners of the inter-company collaborations.

We suspect that similar strategy could also be useful for other countries with similar innovation climate. Of course, quantitative studies of patent records from other countries need to be performed to address this open, yet, interesting question. Particular care should be taken when we deal with some unsuspected factors such as patent data formatting and non-structured social factors. In particular, to understand the impact of collaboration on innovations, we need to consider that different countries have different company climates. For example, Japan and the U.S. have antithetic company climates. The Japanese government regards large companies as important. The Japanese government has promoted indirect financial systems after Second World War and Japanese banks have financed large firms such that they can catch up U.S. technologies. On the contrary, the U.S. government strongly nurtures start-up companies. For example, there is a successful policy called the Small Business Innovations Research (SBIR) that consists of three phases and only successful start-up companies can proceed to the next phase. The policy is the largest innovation policy in U.S. [35]. Based on the above discussion, when we apply the systematic approach proposed in this paper to different dataset obtained from different countries, we need to understand different company climates in those countries first so that we can better understand the impact of collaboration on innovations. We cannot blindly adopt strategies from other countries.

Our systematic approach based on team sizes and repeat collaboration may also be readily applied to other creative projects, such as scientific research [3, 4], consulting practice [5], and entertainment performances [6, 3, 7, 8], to further reveal the intricate relation between collaboration and creativity.

## Supporting Information

S1 Fig Characteristics of collaboration networks. (a-g) Inventors. (h-n) Companies. The collaboration networks of inventors (or companies) can be constructed from the patent records. Naturally, those networks are time varying because of the addition of new patents. Hence, the aggregate statistics of the networks constructed from the whole patent records might not be good measures of the networks constructed at a particular time point. To make sure that the collaboration networks have stabilized to some extent, we calculated seven basic graph characteristics (*N*, *E*, ⟨*k*⟩, *s*_lc_, *n*_0_, *C*, and *r*) of the collaboration networks constructed from the first *p* fraction of the entire patent records under study that are chronologically ordered, with *p* tuned from 0.1 to 1.0. We then plot those graph characteristics as functions of *p*. We found that, though the networks are growing (the number of nodes *N* and number of edges *E* are increasing) and becoming denser (the mean degree ⟨*k*⟩ = 2*E*/*N* is increasing), many other graph characteristics, e.g., the fraction of the largest connected component (*s*_lc_), the fraction of isolated nodes (*n*_0_), the average clustering coefficient (*C*), and the degree correlation (*r*), are actually reaching steady values, especially at the company level.
(TIFF)

S2 Fig Characteristic distributions of collaboration networks. From left to right: Distributions of node degree (*k*), node weight (*w*_n_), link weight (*w*_l_) and component size (*S*). We found that *P*(*k*), *P*(*w*_n_), *P*(*w*_l_), and *P*(*S*), representing the distribution of node degree, node weight, link weight, and component size, respectively, are very stable for *p* > 0.6 at both inventor and company levels. These findings prompt us to compare the stable graph characteristics of the Japanese and U.S. collaboration networks. Surprisingly, we find that those networks show strikingly similar topological features, despite the fact that they cover different years and different number of inventors or companies.
(TIFF)

S3 Fig Impact distributions of patents. Due to the logarithmic scale, the fraction of patents with zero impact value (*P*(*I* = 0)) is not shown: *P*(*I* = 0) = 0.844 for Japan patents and 0.271 for U.S. patents. To quantify the innovation performance of inventors and companies, we need to value the impact of patents they filed. The most commonly used impact measure is the number of citations, which has been verified to be a good indicator of a patent’s impact [27]. Citations were also used to value the performance of other types of creative projects, e.g., scientific papers [25]. To take into account the fact that older patents have a higher chance of being cited, we normalize the number of citations by the average number of citations of patents granted in the same year [11]. We also removed all self-citations to avoid any bias [36]. Hereafter, the impact of patents, denoted as *I*, is defined to be the normalized number of citations. We found both Japanese and U.S. patents show highly heterogeneous impact distributions, implying that most patents have very low impact and only a few patents have huge impact. This might be due to the fact that in U.S. patent applicants have legal duty to cite any prior related patents, while in Japan applicants did not have such legal duty before 2002. Though the patent law in Japan was revised in 2002 to introduce the disclosure duty, we think the effect is rather small on the patent record studied in this work, because 86.7% patent citations occurred before 2002. Moreover, the impact values of Japanese patents have more broader distribution than that of U.S. patents. These results indicate that Japanese patents have higher disparity in impact values than U.S. patents.
(TIFF)

S4 Fig Effects of team productivity on patent impact. (a, b) Inventors. (c, d) Companies. (a, c) Probability distribution of team productivity. (b, d) Patent impact as a function of team productivity. To quantify the innovation experience of a node, we defined the productivity of a node *i* in a patent record to be the number of patents that node *i* has already contributed up to the current patent. We then defined the *team productivity*, denoted as *R*_n_, of a team as the average of all its members’ productivity. For example, in main text Fig. 1 the average productivity of the inventor team in patent-3 is *R*_n_ = (1+2+3)/3 = 2. Note that the repeat collaboration number (*R*_l_) of a team quantifies the accumulated collaboration experience among its team members, which cannot be deduced from the average productivity of the team (*R*_n_). For each patent in the patent records, we calculated the productivity of its inventor team and company team, respectively. We found that at both the inventor and company levels the productivity shows similar distribution for Japan and U.S. patents (see S4 Fig. a, c). At the inventor level, *P*(*R*_n_) shows fat-tail behavior, indicating that many patents are filed by unproductive inventors and only a few patents are filed by highly productive inventors. In contrast, at the company level *P*(*R*_n_) shows an almost uniform distribution for *R*_n_ up to 10^4^, implying that company teams of a wide range of productivity contribute equally to innovations. The drastic drop of *P*(*R*_n_) as *R*_n_ > 10^4^ for both Japanese and U.S. companies suggests a natural upper bound of innovation productivity at the company level. We then calculated the average impact for patents of similar productivity grouped in logarithmic bins (see S4 Fig. b, d). At the inventor level, Japan and U.S. patents display quite different behaviors. For Japanese inventors, their innovation performance improves first as *R*_n_ increases, reaches its peak value around *R*_n_ ∼ 40, and then generally degrades. In contrast, the performance of U.S. inventors degrades almost monotonically as *R*_n_ increases. At the company level, both Japan and U.S. companies display relatively stable behavior for productivity *R*_n_ up to 10^3^. Note that repetitions of joint-application by company teams are probably conducted by different inventors. The stability of company innovation performance by different inventors are subject to organizational influence. We also observed that the innovation performance of U.S. companies slightly improves as *R*_n_ > 10^3^ until a dramatic performance degradation as *R*_n_ > 10^4^. Since the fraction of patents with *R*_n_ > 10^4^ is rather small (≤ 0.01%), such a degradation could be due to statistical noise.
(TIFF)

S5 Fig Effects of team age on patent impact. (a, b) Inventors. (c, d) Companies. (a, c) Probability distribution of team age. (b, d) Patent impact as a function of team age. We also defined *team age*, denoted as *A*, by averaging its team member’s “age”. Here, the “age” of a node *i* in a patent record is defined to be the duration from its first application year to the application year of the current patent. Note that the team age is not necessarily related to collaboration, because a very old team could be just due to its team members are very old but not very productive and/or collaborative at all. For each patent in the patent records, we calculated the age of its inventor team and company team, respectively. At the inventor level, *P*(*A*) is very heterogeneous, implying that most patents are invented by “young” inventors with small *A* and only a few patents are invented by “old” inventors with large *A*. In contrast, at the company level *P*(*A*) shows two strong peaks: (1) *A* = 0 for both U.S. and Japan teams; (2) *A* ≈ 8 for Japan teams or *A* ≈ 13 for U.S. teams. We found that Japanese inventor teams and U.S. inventor teams have roughly the same age distribution (see S5 Fig. a). Yet, U.S. company teams and Japanese company teams have quite different age distributions (see S5 Fig. c). We then calculated the average impact for patents of the same age (see S5 Fig. b, d). We found that the team performance behaves differently at the two different levels as the team age increases. At the inventor level, both Japanese and U.S. inventor teams’ performance degrades gradually as team age *A* increases. Similar trend is observed in Japan company teams. Yet, we found that U.S. company teams show quite stable performance as *A* increases. Note that for both inventor and company teams, their performance displays large fluctuations for teams with very large *A*, which could be due to the fact that “old” teams are very rare in both Japan and U.S. patent records (see S5 Fig. a, c).
(TIFF)

S1 Table Variables for regression analyses. (TIFF)

S2 Table Correlation matrix of Japan-inventor. (TIFF)

S3 Table Correlation matrix of US-inventor. (TIFF)

S4 Table Correlation matrix of Japan-company. (TIFF)

S5 Table Correlation matrix of US-company. (TIFF)

S6 Table Regression result of Japan-inventor. (TIFF)

S7 Table Regression result of US-inventor. (TIFF)

S8 Table Regression result of Japan-company. (TIFF)

S9 Table Regression result of US-company. (TIFF)

S10 Table Result of automatic variable selection by LASSO. (TIFF)
