# Supplementary figures and images for: Revealing the Intricate Effect of Collaboration on Innovation
Source: PLoS One. 2015 Mar 23;10(3):e0121973. doi: 10.1371/journal.pone.0121973 (PMC4370822; doi:10.1371/journal.pone.0121973)

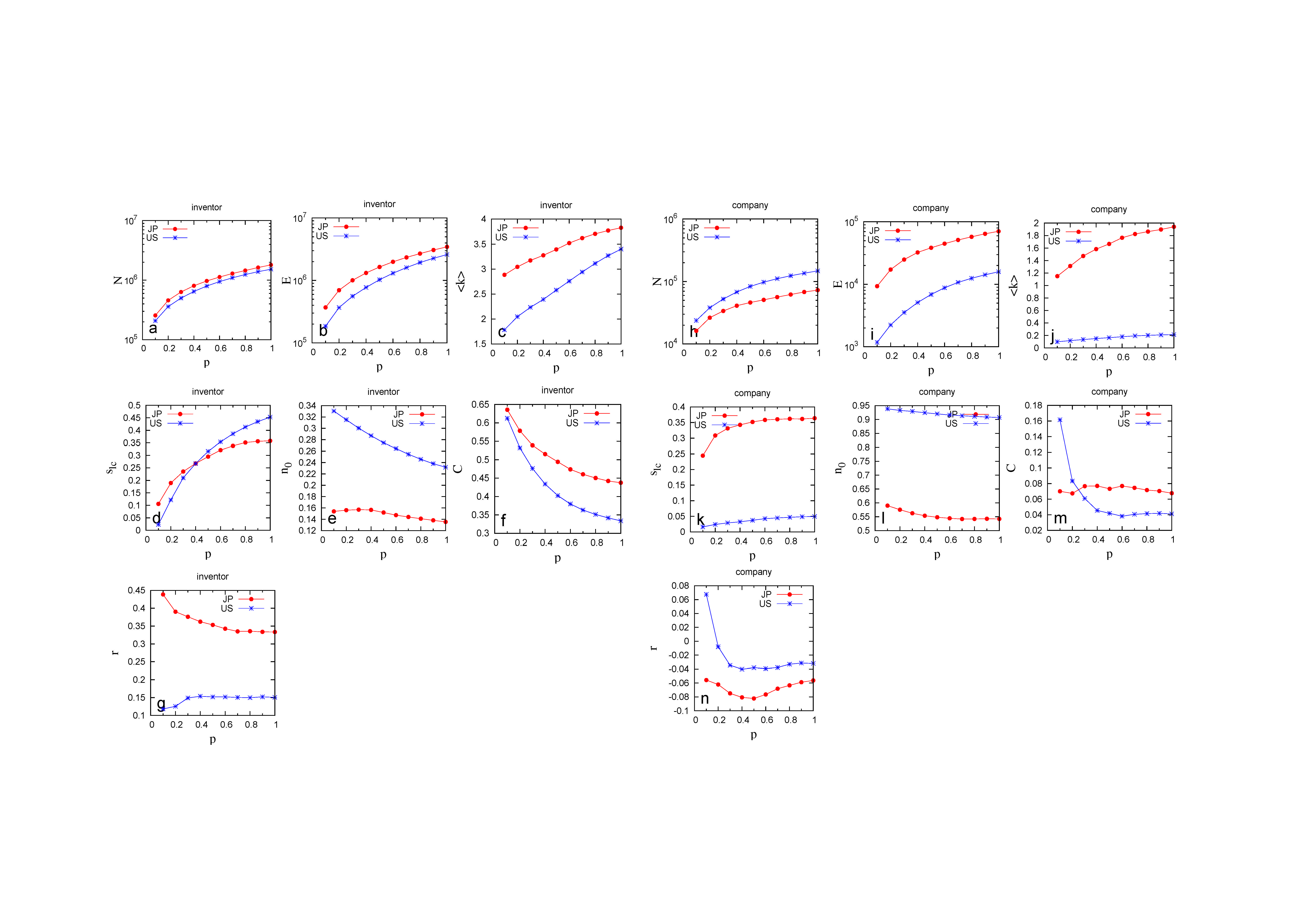

Supplement: S1 Fig — The collaboration networks of inventors (or companies) can be constructed from the patent records. Naturally, those networks are time varying because of the addition of new patents. Hence, the aggregate statistics of the networks constructed from the whole patent records might not be good measures of the networks constructed at a particular time point. To make sure that the collaboration networks have stabilized to some extent, we calculated seven basic graph characteristics (N, E, ⟨k⟩, s lc, n 0, C, and r) of the collaboration networks constructed from the first p fraction of the entire patent records under study that are chronologically ordered, with p tuned from 0.1 to 1.0. We then plot those graph characteristics as functions of p. We found that, though the networks are growing (the number of nodes N and number of edges E are increasing) and becoming denser (the mean degree ⟨k⟩ = 2E/N is increasing), many other graph characteristics, e.g., the fraction of the largest connected component (s lc), the fraction of isolated nodes (n 0), the average clustering coefficient (C), and the degree correlation (r), are actually reaching steady values, especially at the company level. (TIFF) [file pone.0121973.s001.tiff]

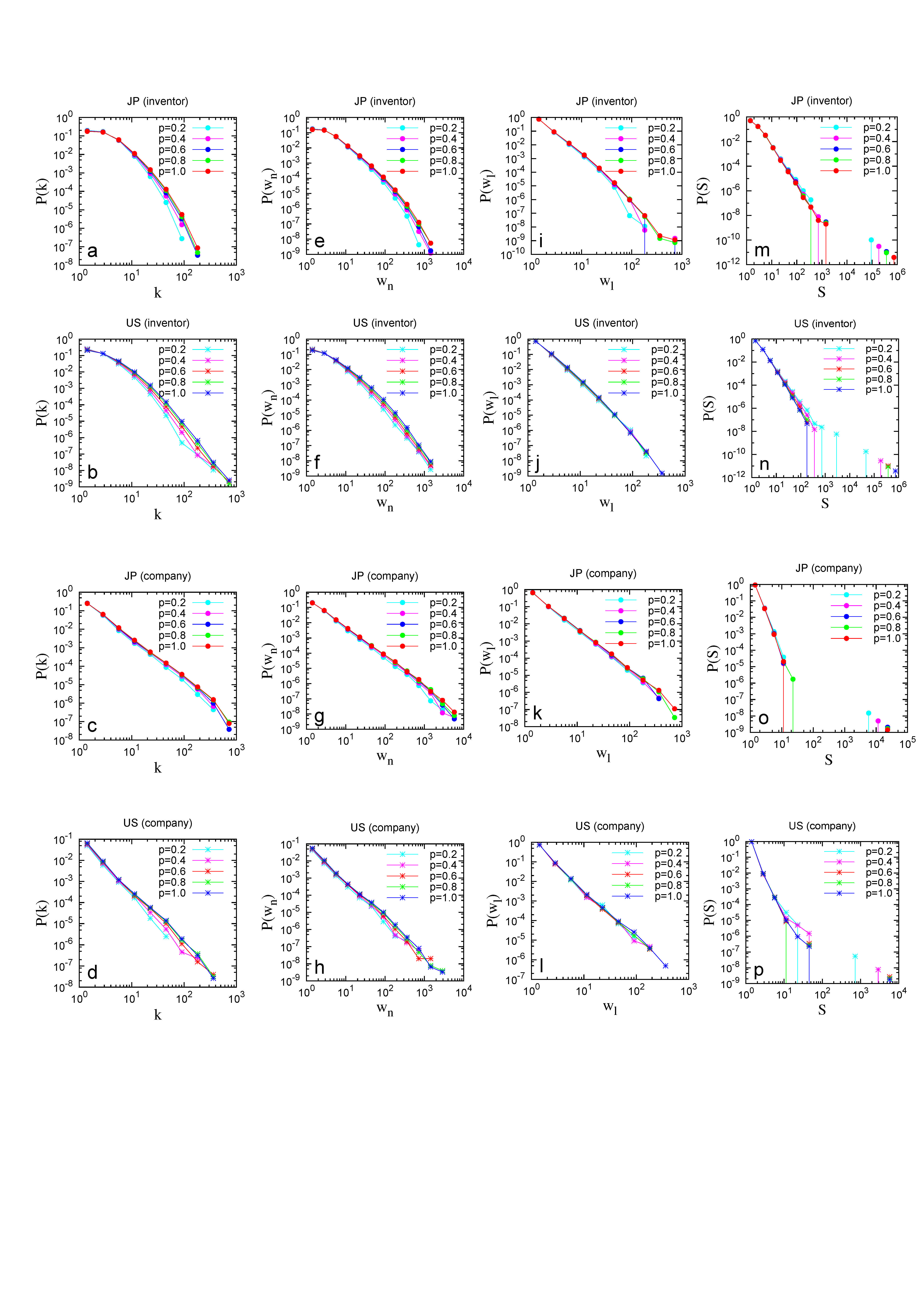

Supplement: S2 Fig — We found that P(k), P(w n), P(w l), and P(S), representing the distribution of node degree, node weight, link weight, and component size, respectively, are very stable for p > 0.6 at both inventor and company levels. These findings prompt us to compare the stable graph characteristics of the Japanese and U.S. collaboration networks. Surprisingly, we find that those networks show strikingly similar topological features, despite the fact that they cover different years and different number of inventors or companies. (TIFF) [file pone.0121973.s002.tiff]

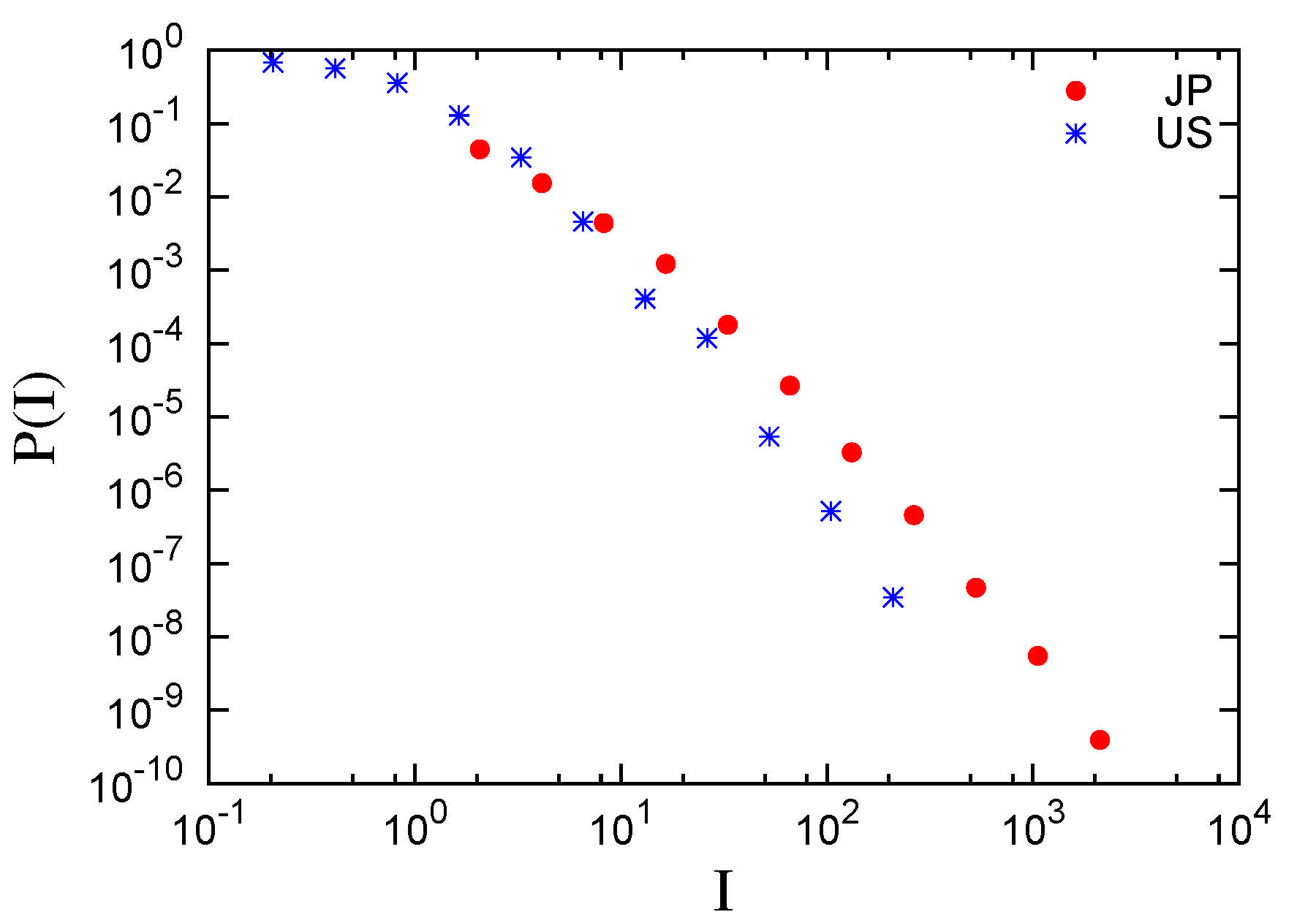

Supplement: S3 Fig — To quantify the innovation performance of inventors and companies, we need to value the impact of patents they filed. The most commonly used impact measure is the number of citations, which has been verified to be a good indicator of a patent’s impact [27]. Citations were also used to value the performance of other types of creative projects, e.g., scientific papers [25]. To take into account the fact that older patents have a higher chance of being cited, we normalize the number of citations by the average number of citations of patents granted in the same year [11]. We also removed all self-citations to avoid any bias [36]. Hereafter, the impact of patents, denoted as I, is defined to be the normalized number of citations. We found both Japanese and U.S. patents show highly heterogeneous impact distributions, implying that most patents have very low impact and only a few patents have huge impact. This might be due to the fact that in U.S. patent applicants have legal duty to cite any prior related patents, while in Japan applicants did not have such legal duty before 2002. Though the patent law in Japan was revised in 2002 to introduce the disclosure duty, we think the effect is rather small on the patent record studied in this work, because 86.7% patent citations occurred before 2002. Moreover, the impact values of Japanese patents have more broader distribution than that of U.S. patents. These results indicate that Japanese patents have higher disparity in impact values than U.S. patents. (TIFF) [file pone.0121973.s003.tiff]

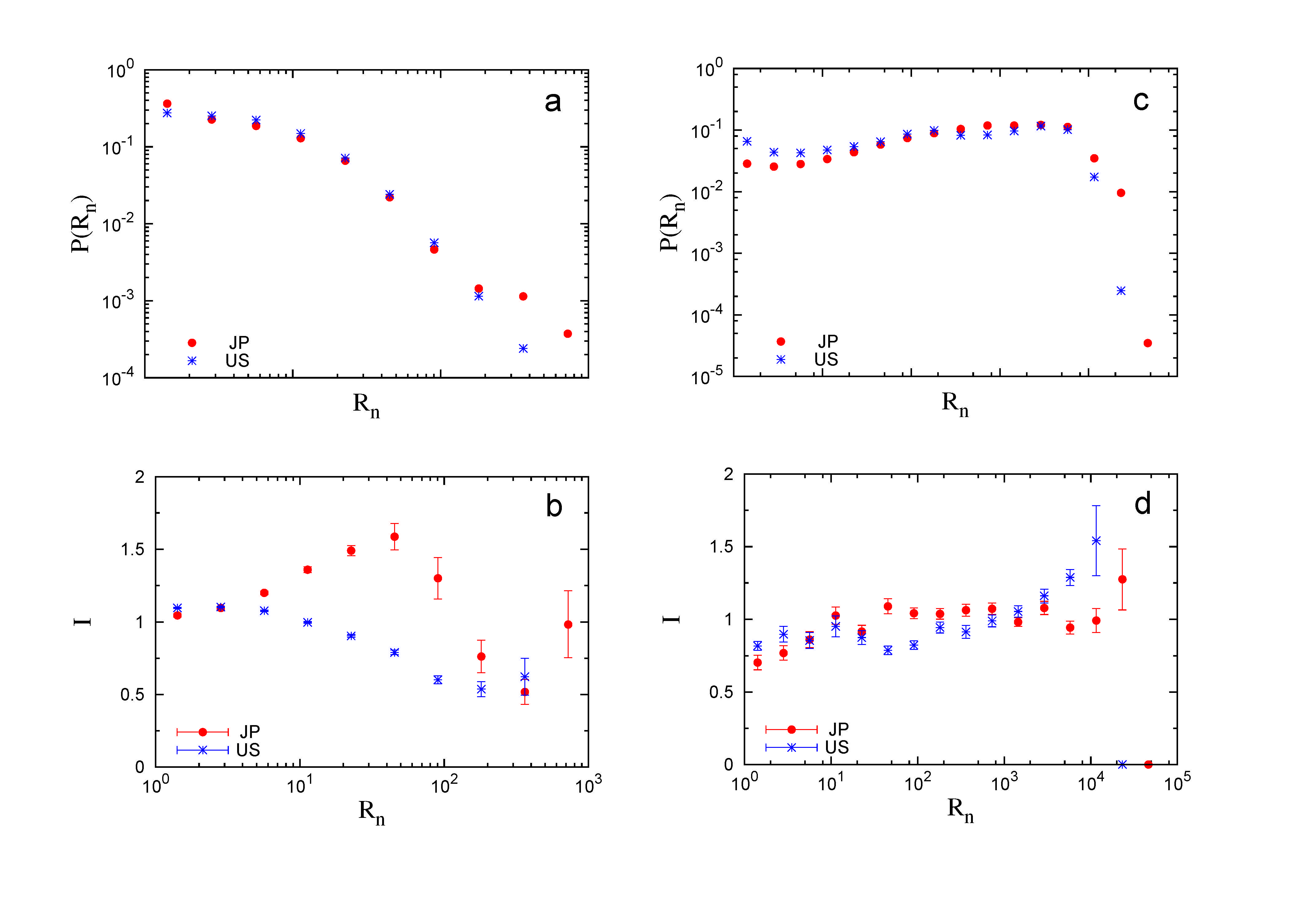

Supplement: S4 Fig — To quantify the innovation experience of a node, we defined the productivity of a node i in a patent record to be the number of patents that node i has already contributed up to the current patent. We then defined the team productivity, denoted as R n, of a team as the average of all its members’ productivity. For example, in main text Fig. 1 the average productivity of the inventor team in patent-3 is R n = (1+2+3)/3 = 2. Note that the repeat collaboration number (R l) of a team quantifies the accumulated collaboration experience among its team members, which cannot be deduced from the average productivity of the team (R n). For each patent in the patent records, we calculated the productivity of its inventor team and company team, respectively. We found that at both the inventor and company levels the productivity shows similar distribution for Japan and U.S. patents (see S4 Fig. a, c). At the inventor level, P(R n) shows fat-tail behavior, indicating that many patents are filed by unproductive inventors and only a few patents are filed by highly productive inventors. In contrast, at the company level P(R n) shows an almost uniform distribution for R n up to 104, implying that company teams of a wide range of productivity contribute equally to innovations. The drastic drop of P(R n) as R n > 104 for both Japanese and U.S. companies suggests a natural upper bound of innovation productivity at the company level. We then calculated the average impact for patents of similar productivity grouped in logarithmic bins (see S4 Fig. b, d). At the inventor level, Japan and U.S. patents display quite different behaviors. For Japanese inventors, their innovation performance improves first as R n increases, reaches its peak value around R n ∼ 40, and then generally degrades. In contrast, the performance of U.S. inventors degrades almost monotonically as R n increases. At the company level, both Japan and U.S. companies display relatively stable behavior for productivity R n up [file pone.0121973.s004.tiff]

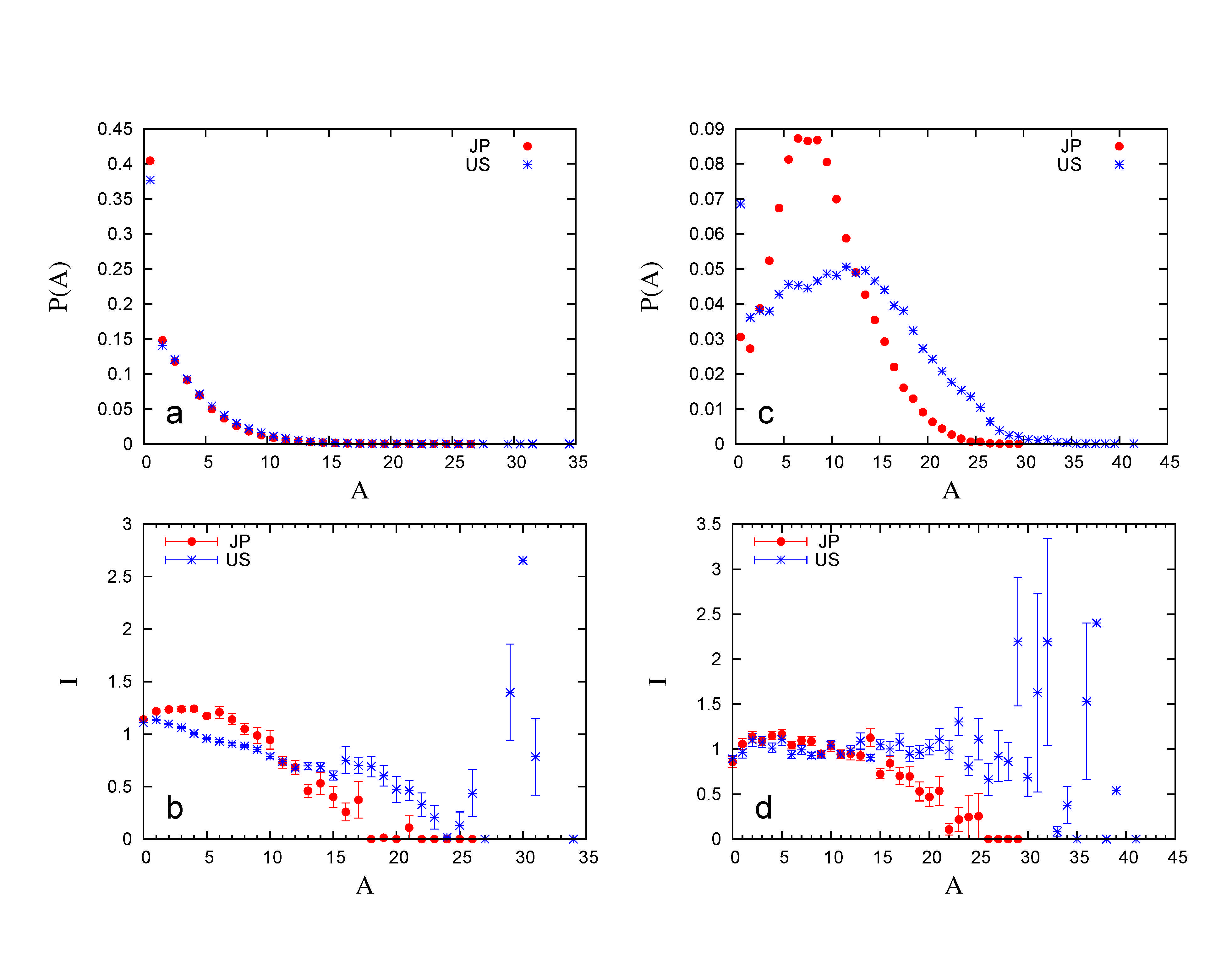

Supplement: S5 Fig — We also defined team age, denoted as A, by averaging its team member’s “age”. Here, the “age” of a node i in a patent record is defined to be the duration from its first application year to the application year of the current patent. Note that the team age is not necessarily related to collaboration, because a very old team could be just due to its team members are very old but not very productive and/or collaborative at all. For each patent in the patent records, we calculated the age of its inventor team and company team, respectively. At the inventor level, P(A) is very heterogeneous, implying that most patents are invented by “young” inventors with small A and only a few patents are invented by “old” inventors with large A. In contrast, at the company level P(A) shows two strong peaks: (1) A = 0 for both U.S. and Japan teams; (2) A ≈ 8 for Japan teams or A ≈ 13 for U.S. teams. We found that Japanese inventor teams and U.S. inventor teams have roughly the same age distribution (see S5 Fig. a). Yet, U.S. company teams and Japanese company teams have quite different age distributions (see S5 Fig. c). We then calculated the average impact for patents of the same age (see S5 Fig. b, d). We found that the team performance behaves differently at the two different levels as the team age increases. At the inventor level, both Japanese and U.S. inventor teams’ performance degrades gradually as team age A increases. Similar trend is observed in Japan company teams. Yet, we found that U.S. company teams show quite stable performance as A increases. Note that for both inventor and company teams, their performance displays large fluctuations for teams with very large A, which could be due to the fact that “old” teams are very rare in both Japan and U.S. patent records (see S5 Fig. a, c). (TIFF) [file pone.0121973.s005.tiff]

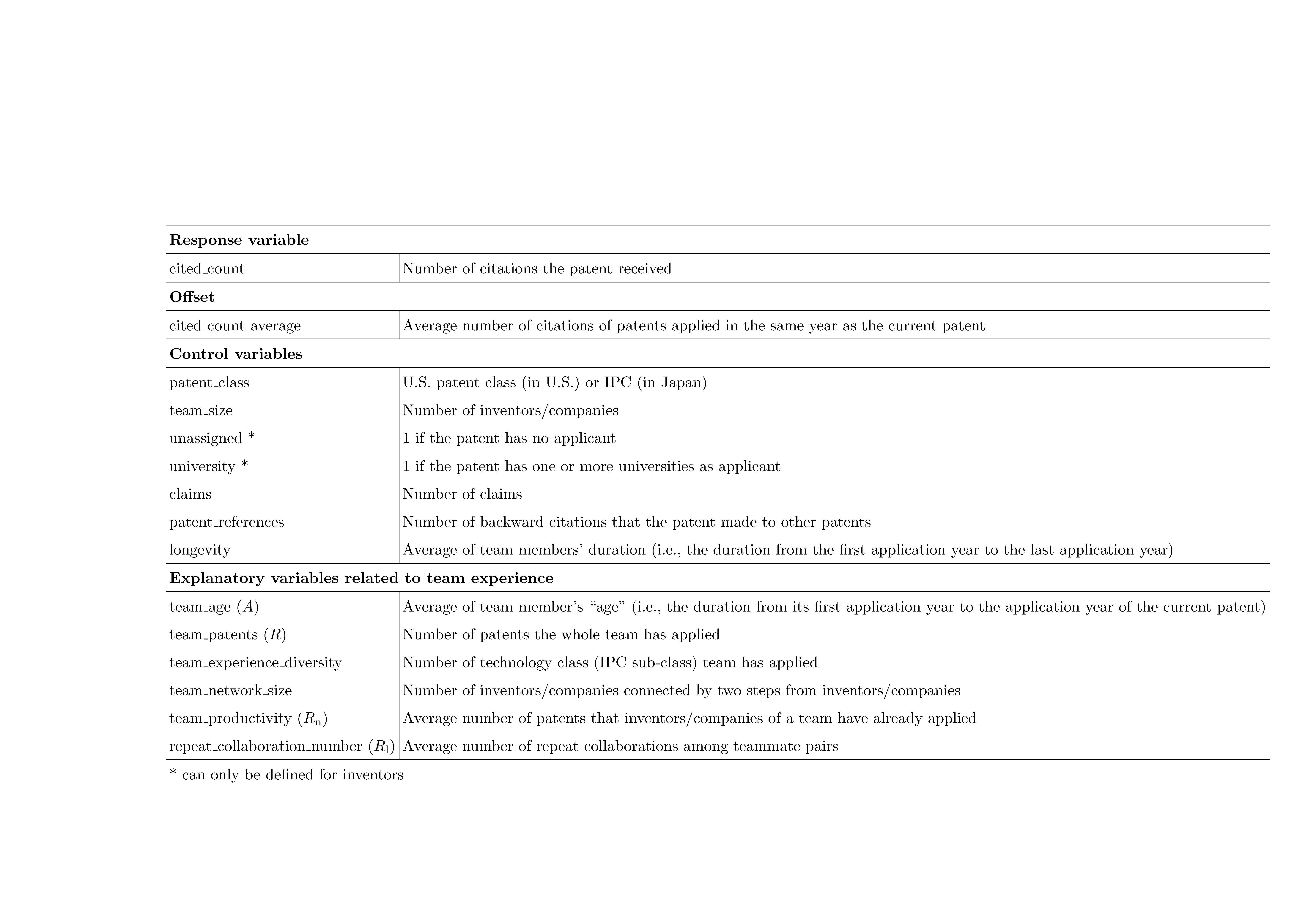

Supplement: S1 Table — (TIFF) [file pone.0121973.s006.tiff]

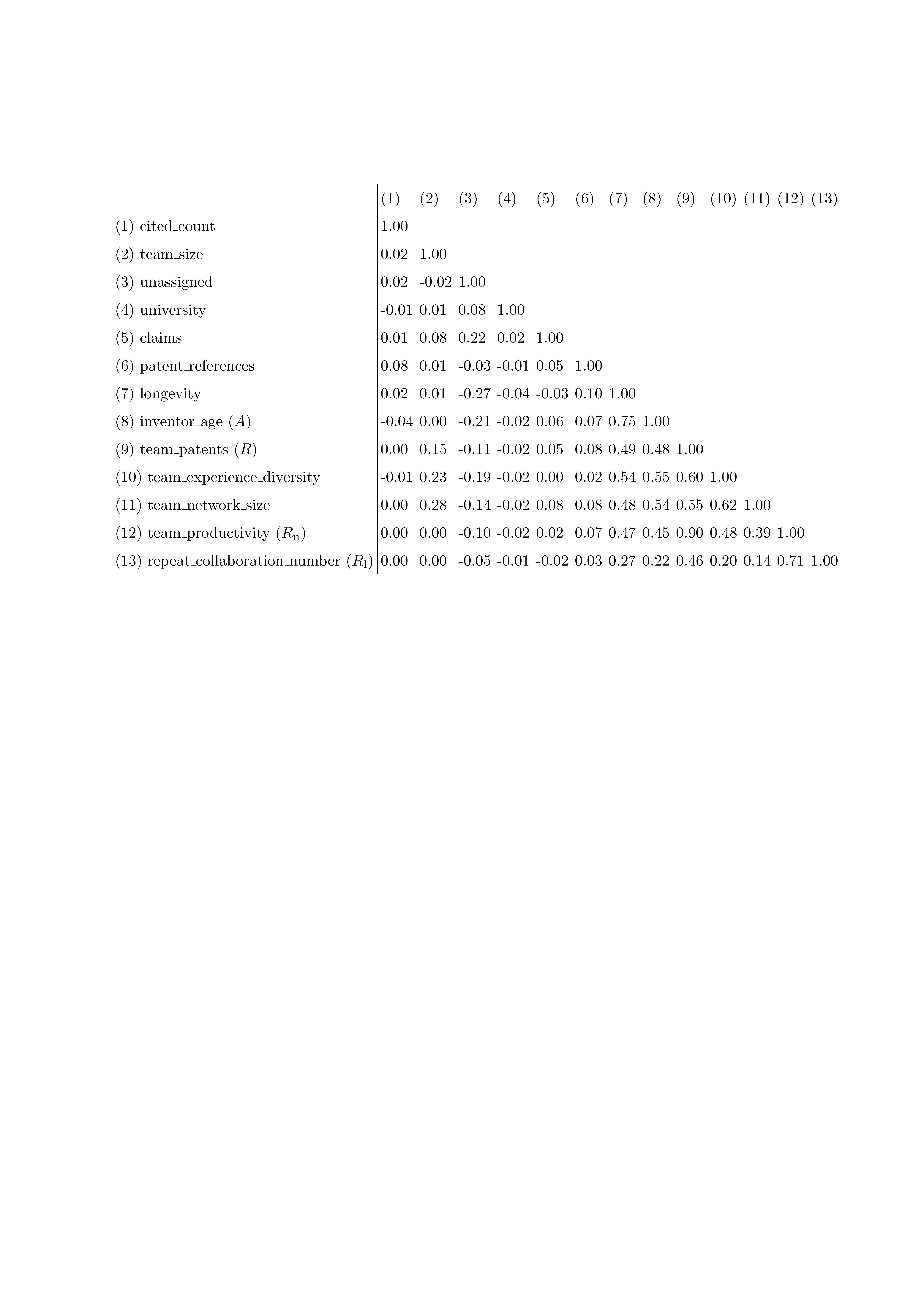

Supplement: S2 Table — (TIFF) [file pone.0121973.s007.tiff]

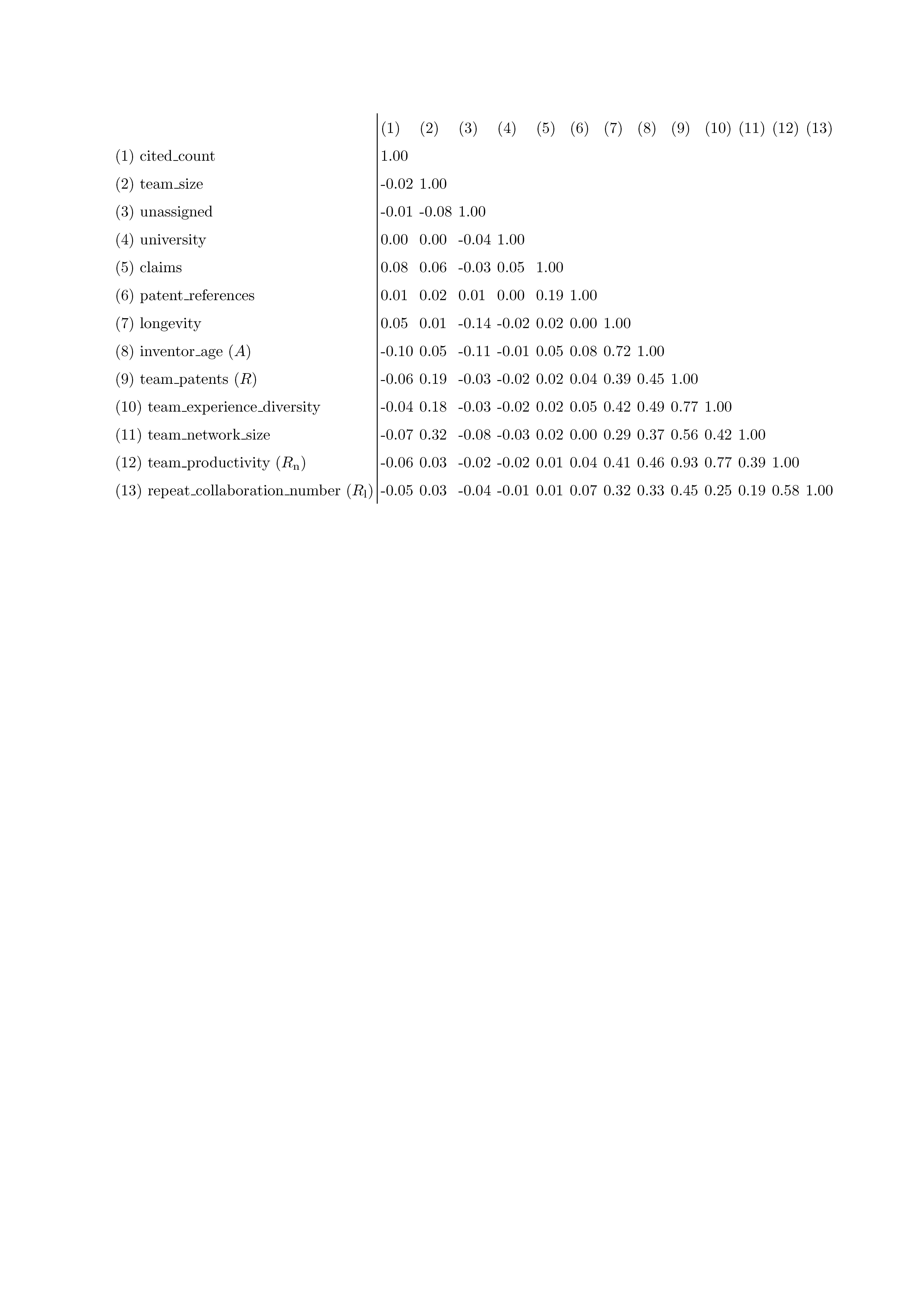

Supplement: S3 Table — (TIFF) [file pone.0121973.s008.tiff]

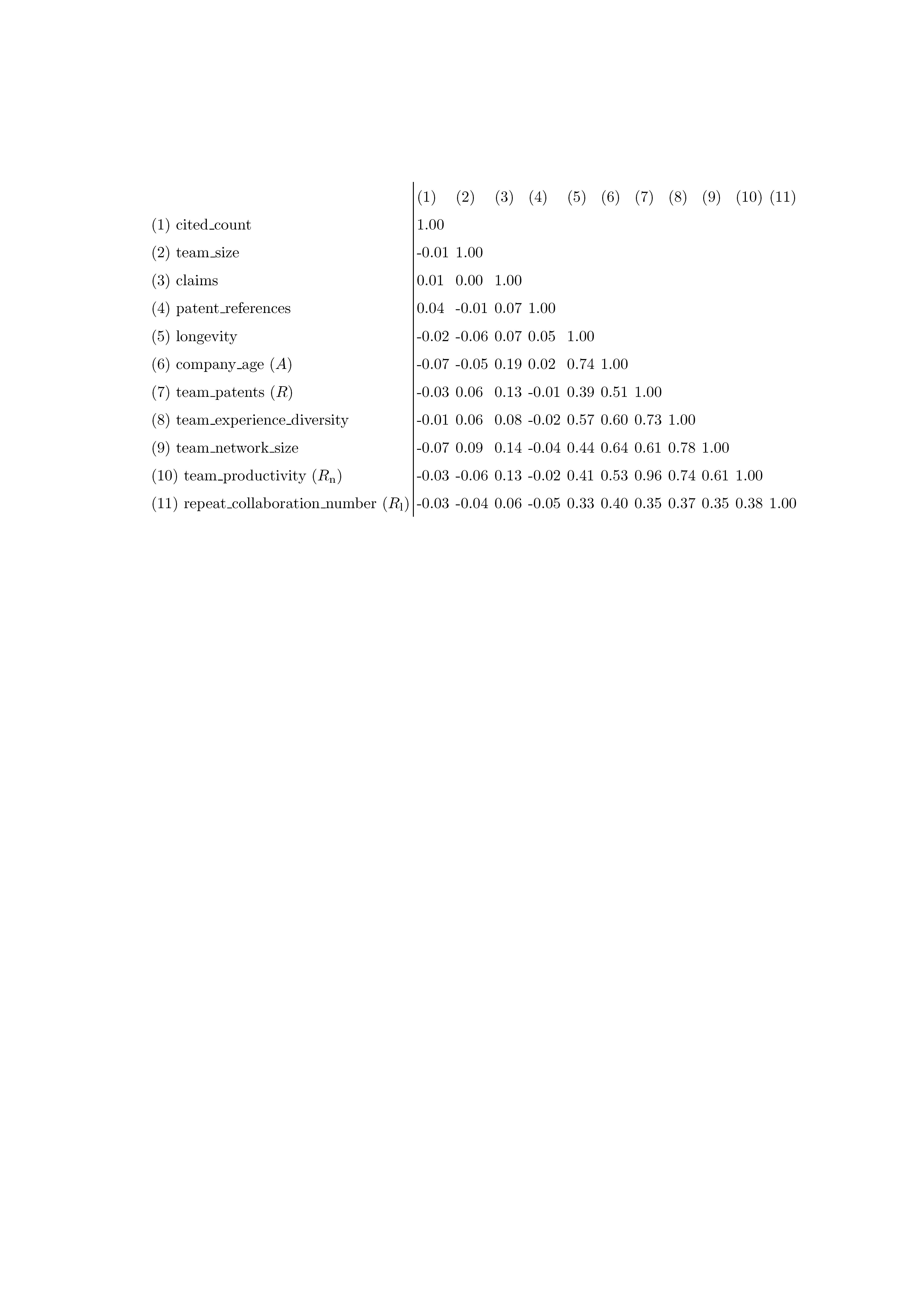

Supplement: S4 Table — (TIFF) [file pone.0121973.s009.tiff]

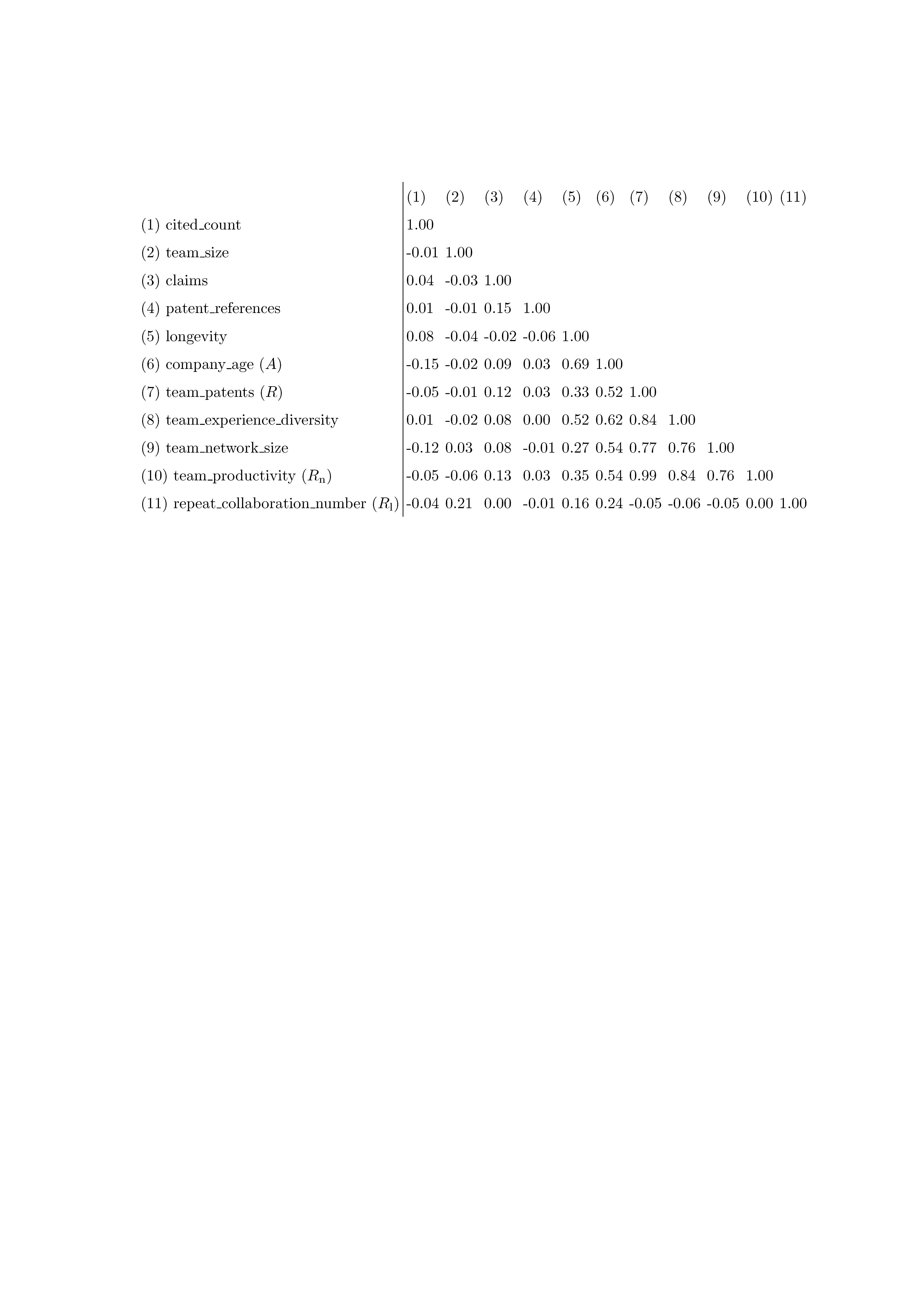

Supplement: S5 Table — (TIFF) [file pone.0121973.s010.tiff]

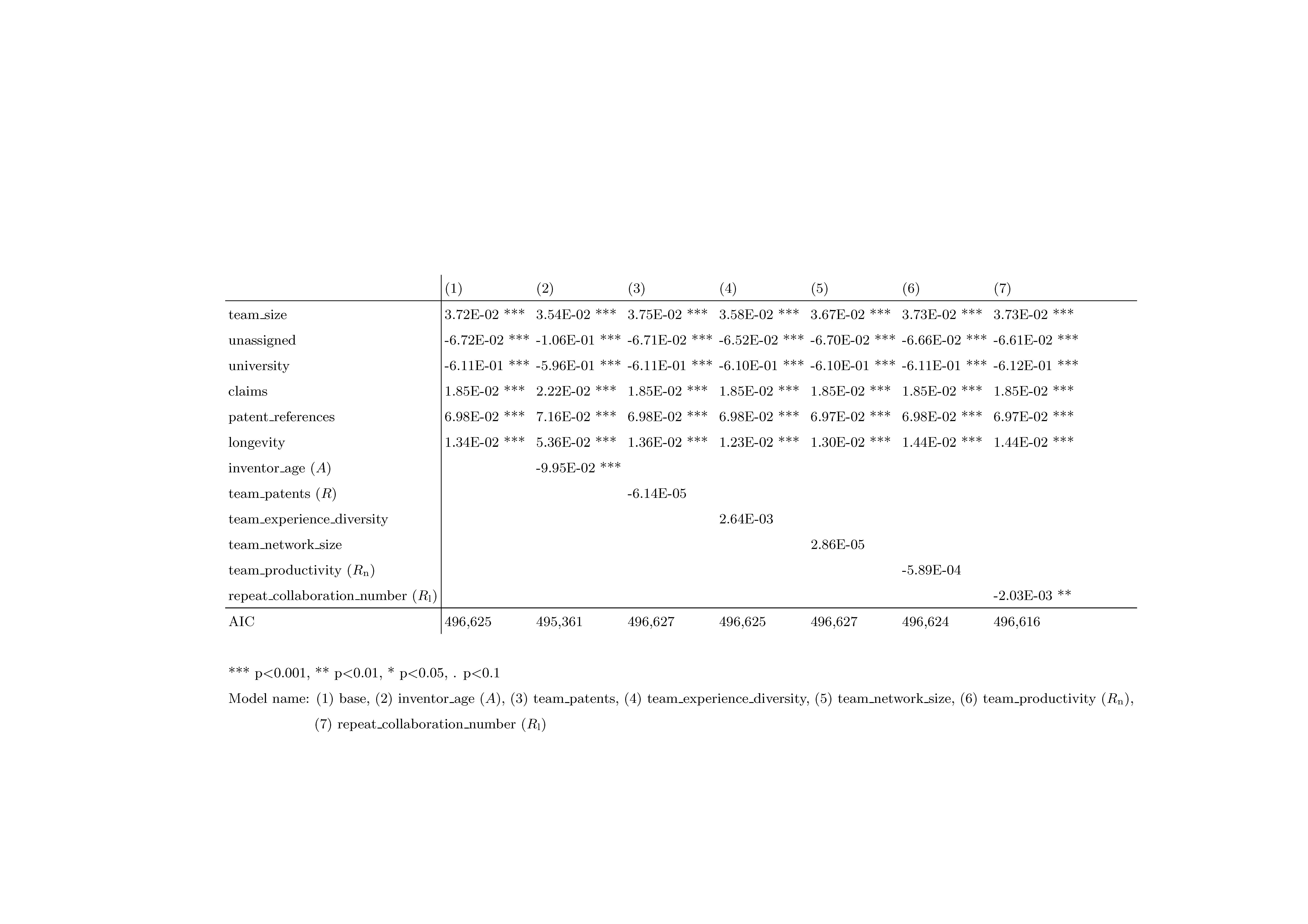

Supplement: S6 Table — (TIFF) [file pone.0121973.s011.tiff]

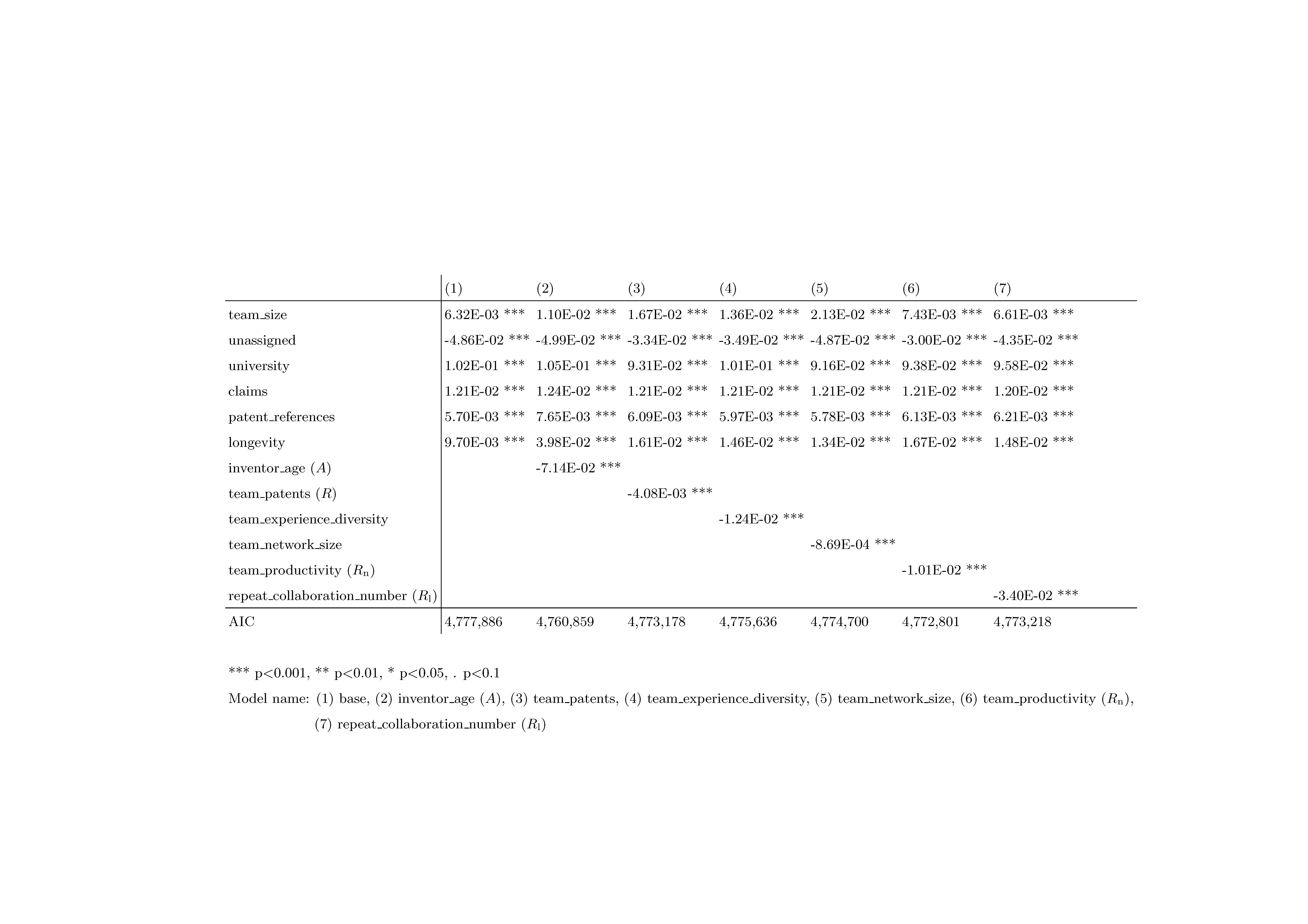

Supplement: S7 Table — (TIFF) [file pone.0121973.s012.tiff]

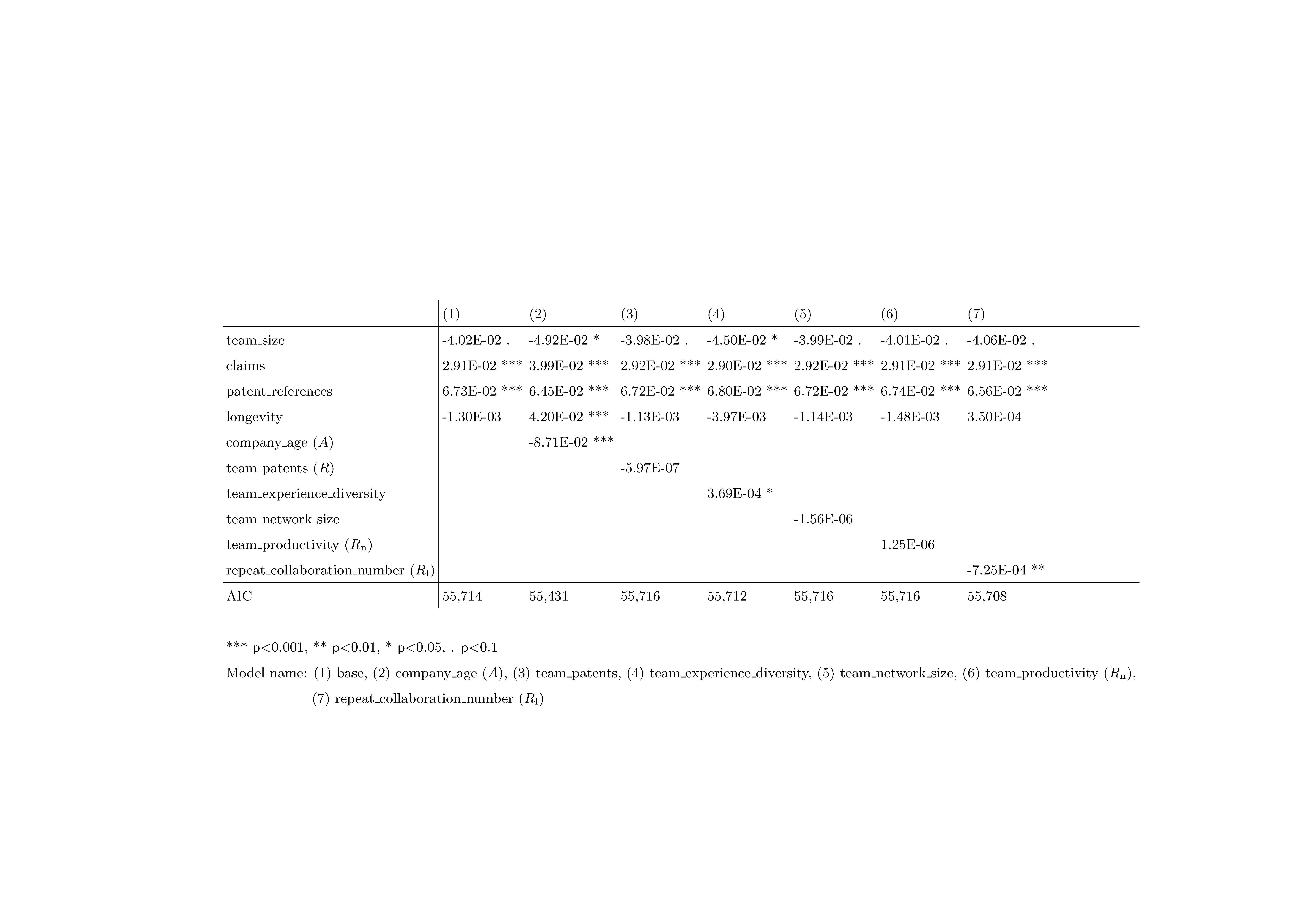

Supplement: S8 Table — (TIFF) [file pone.0121973.s013.tiff]

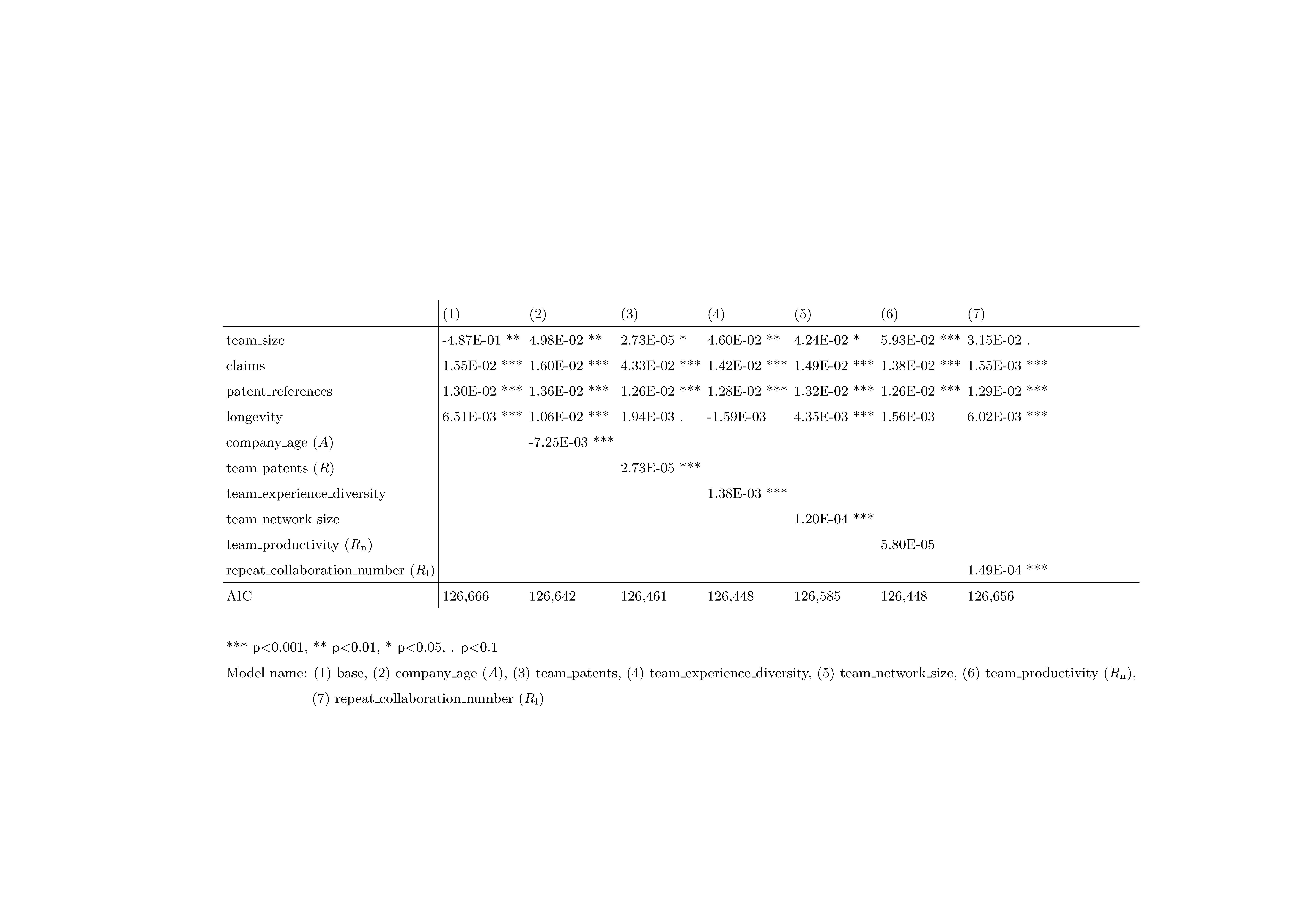

Supplement: S9 Table — (TIFF) [file pone.0121973.s014.tiff]

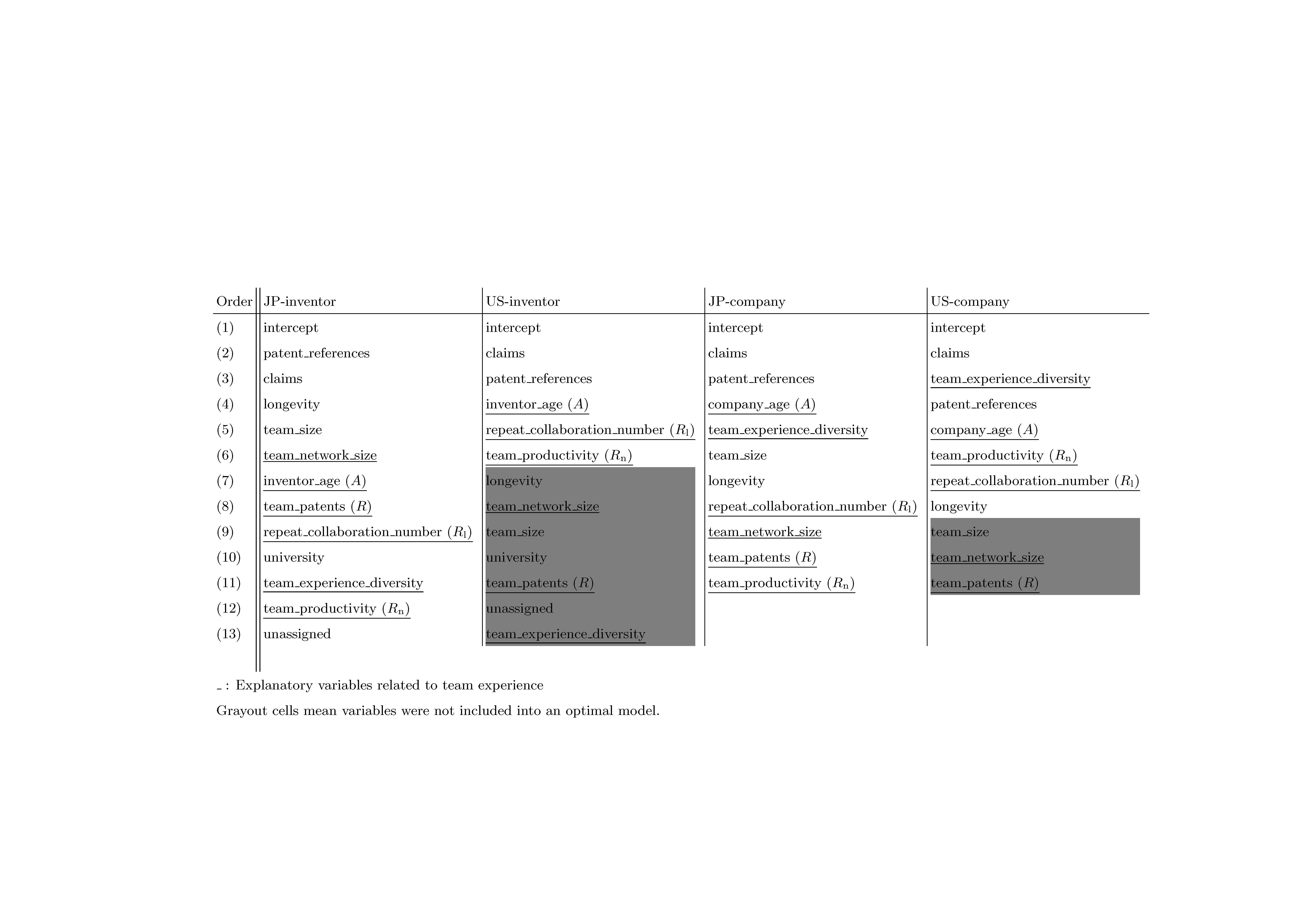

Supplement: S10 Table — (TIFF) [file pone.0121973.s015.tiff]
